# Laurence-Moon-Bardet-Biedl Syndrome with Coexisting Abdominal Distension and Positive Fluid Thrill: A Rare Manifestation Reported in Karachi, Pakistan

**DOI:** 10.7759/cureus.4885

**Published:** 2019-06-11

**Authors:** Laila Tul Qadar, Zohaib M Ahmed, Maham Munawar, Choudhary A Hasan, Syed Umair Iqbal

**Affiliations:** 1 Internal Medicine, Dow University of Health Sciences (DUHS), Karachi, PAK

**Keywords:** bardet-biedl syndrome, chronic kidney disease, retinitis pigmentosa, polydactyly, central obesity, consanguineous marriage, congenital disorder, positive fluid thrill, abdominal distension

## Abstract

Laurence-Moon-Bardet-Biedl syndrome (LMBBS) is a rare autosomal recessive (AR) disorder. It is characterized predominantly by hypogonadism, polydactyly, retinitis pigmentosa, obesity, and mental retardation. Herein, we present a classic case of LMBBS with generalized body edema, abdominal distension, and positive fluid thrill in a 32-year-old male. LMBBS patients are friendly with a happy predisposition, proper management, and regular examinations should be done in order to maintain healthy organ function and to avoid an early death. Renal failure is the most common cause of mortality in LMBBS patients.

## Introduction

Laurence-Moon-Bardet-Biedl syndrome (LMBBS) is a rare autosomal recessive (AR) disorder associated with five fundamental characteristics including retinitis pigmentosa, polydactyly, obesity, and hypogonadism and mental retardation. Laurence-Moon syndrome (LMS) and Bardet-Biedl syndrome (BBS) are usually referred to as a single syndrome but these are two distinct entities.

Both syndromes are genetically inherited and are AR. However, LMS has pigmentary retinopathy along with hypogonadism, mental retardation and neurological defects accompanied by spastic paraplegia but no polydactyly or obesity, which are the key features in BBS. Congenital heart diseases and nephropathy are more pronounced in BBS. Despite many obvious symptoms, the syndrome continues to remain an underdiagnosed condition. It has a slight female predominance and less than half the affected patients are males. The occurrence is 1:140000 to 1:160000 in North America and Europe respectively, while greater occurrence was reported in Kuwait and Newfoundland, having 1:13500 to 1:17500 respectively [1]. Renal function loss is established as the root of a high mortality rate [2]. Here in we report a case of an LMBBS patient presented with abdominal distension and positive fluid thrill. Following clinical presentations are often rare to present with this syndrome. This case, therefore, highlights the possibility of a wide range of symptoms presenting with LMBBS.

## Case presentation

A 32-year-old male presented to medicine outpatient department (OPD) of Dr. Ruth KM Pfau, Civil Hospital Karachi (CHK) with the complaint of abdominal distension for 1.5 months. The patient was in his usual state of health before that. Following his admission as we looked into his illness the abdominal distension was soft and non-tender with positive fluid thrill, on examination, his respiratory rate (RR) was 18 breaths/min, blood pressure (BP) was 110/90 mmHg and a pulse of 78 beats/min. It negated the unusual bowel associations. He had pedal edema for two months and there was generalized body edema with periorbital puffiness.

**Figure 1:**
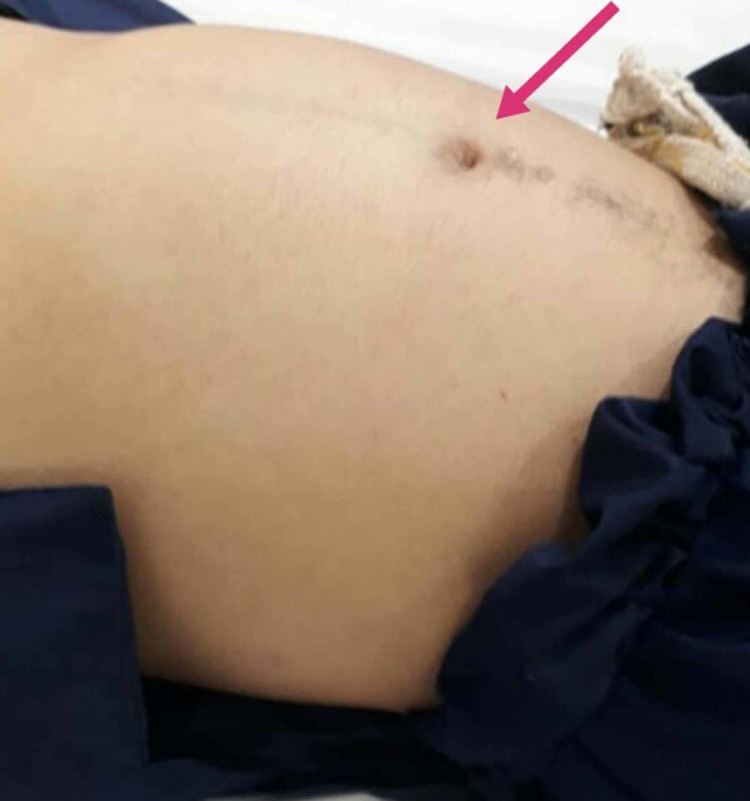
Distended abdomen with slightly everted umbilicus

Upon examination, he appeared to be a young man of average height and obese structure with right hand polydactyly and syndactyly with low intelligence quotient (IQ). He lost his vision at three years of age which started off with night blindness and an ophthalmology consult confirmed retinitis pigmentosa. He has also had an impairment of speech since the first decade of life. The targeted physical examination confirmed the diagnosis of LMBBS where we discovered gynecomastia and bilateral atrophic testes measuring 2.0x1.2 cm right side and 2.2x0.9 cm left side located in inguinal canals, encysted hydrocele was also noted on both sides. Detailed physical examination revealed abdominal distension (Figure 1), fluid thrill, periorbital puffiness, leukonychia, and pedal edema. His vitals were normal. He had increased sleep, appetite, normal bowel habits but decreased mobility and was usually confined to his bed. He has delayed developmental history and has been intellectually impaired since the age of five years. Glasgow coma scale (GCS) was 15/15. All limb movements were intact however he was dependent on others for self-care. Past history also disclosed that he had a urethral stricture in 2011 which was managed with a cystostomy.

**Figure 2:**
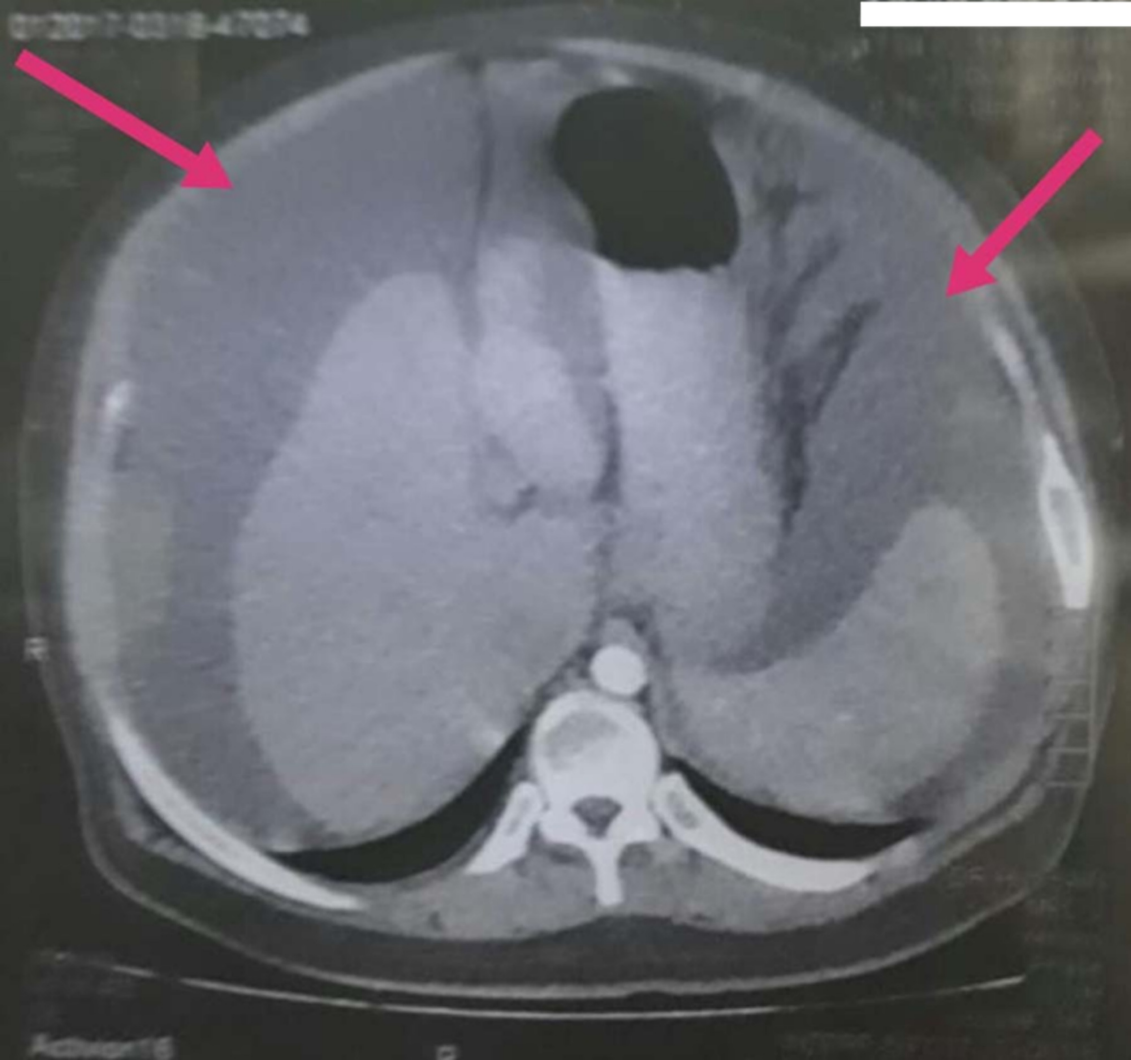
Axial view CT abdomen, periphery showing ascites CT: Computed tomography

**Figure 3:**
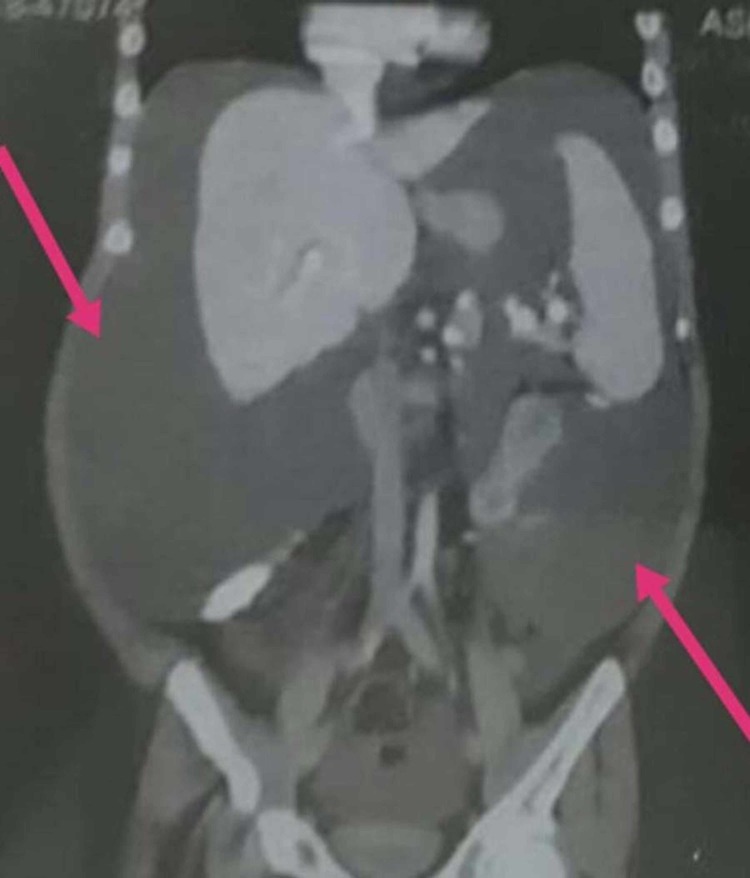
Coronal view CT abdomen showing ascites more marked in pelvic region CT: Computed tomography

The computed tomography (CT) scan report showed ascites in the abdomen and pelvic region (Figures 2-3), and generalized body edema. Liver function tests (LFTs), CT scan and ascitic fluid drainage ruled out chronic liver disease (CLD) and abdominal tuberculosis (TB). Doppler scan of the portal vein was unremarkable as well. Serum testosterone levels were very low at 10.93 ng/dL (Normal (N) = 270-1070), for which supplements were prescribed. Family history revealed that the patient was a product of a consanguineous marriage. He has six siblings with no illness. His mother had TB two years back, which was treated.

To evaluate his abdominal distension a thorough laboratory workup was performed. A complete blood count (CBC) turned out to be completely normal with hemoglobin (Hb) of 11.3 g/dL (N= 13.5-17.5), red blood cell (RBC) count of 4.41x103 million/μl, platelet count of 199x103/μl and a total leukocyte count (TLC) count of 6.2x103/μl. LFTs and serum electrolytes were in a normal range. Ascitic fluid revealed a protein count of 5.6 mg/dl, amylase of 43 IU/l, RBC count of 1000/μl, leukocyte count of 87 and a white blood cell (WBC) count of 354/μl. Urine detailed report showed a rise in WBC count of around 9 cells/HPF (N = less than 2-5). A comprehensive endocrinology workup showed low levels of serum testosterone, elevated follicle stimulating hormone (FSH) of 47.64 IU/l (N = 1-10 IU/l), raised prolactin levels of 27.72 ng/ml (N = 2-20 ng/ml) and an elevated luteinizing hormone (LH) of 29.65 IU/l (N = 0.6-12). However, lipid profile was completely in range.

## Discussion

The patient described in this case report is a typical case of LMBBS, with an unusual presentation of abdominal distension with positive fluid thrill. Genetic techniques have enabled the scientists to identify the responsible genes and as of now, there are 19 genes associated with this disease, all of which encode proteins for cilia [3]. LMBBS is observed in children of consanguineous marriage like all other AR diseases that tend to run in families. It is vital for both parents to carry one copy of the mutated gene for the child to develop LMBBS; even so, there is only a 25 % chance of inheriting the disease. It has been stated that the average age for diagnosis is nine years, albeit there are cases where the patient was found to be 50 years old at the time of diagnosis. Our patient was 32 years old at the time of his diagnosis [4].

The first prime feature of LMBBS is retinal dystrophy, found in the first decade of life in a few people and by the second decade, it is observed in all [5]. Obesity begins in childhood and progresses as the patient ages, with a frequency of 72-96 % as determined by the measurement criteria. The average body mass index (BMI) reported in female patients is 31.5 kg/m2, while in male patients is 36.6 kg/m2 [5-6]. Hypogonadism is more dominant in BBS male patients than females [7]. Owing to this reason, males are infertile in most cases, with the exception of a few patients who successfully got married and were able to have children [4]. Limb abnormalities are another main feature of this syndrome, polydactyly is found in 69% of LMBBS patients but its absence should not be the reason to disregard the diagnoses [4].

Mental retardation is debatable but most patients have lower than normal IQ [6]. Our patient presented with all five aforementioned fundamental symptoms along with renal dysfunction. In 1999 a modified diagnostic criterion was devised by Beales et al., after a study was done on 109 BBS patients [4]. According to the criteria, a patient should have at least four primary features or three primary and two secondary features to be identified as a BBS patient. Our patient has all six primary (Table 1) and four secondary features (Table 2).

**Table 1:** Modified diagnostic criteria for BBS representing primary features BBS: Bardet-Biedl syndrome

Primary features	Present (+) or absent (-)
Rod cone dystrophy	+
Polydactyly	+
Obesity	+
Learning disabilities	+
Hypogonadism in males	+
Renal Anomalies	+

**Table 2:** Modified diagnostic criteria for BBS representing secondary features BBS: Bardet-Biedl syndrome; LVH: Left ventricular hypertrophy

Secondary features	Present (+) or absent (-)
Speech disorder/delay	+
Strabismus/cataract/astigmatism	+
Brachydactyly/syndactyly	+
Developmental delay	+
Polyuria/Polydipsia	-
Ataxia/poor coordination/imbalance	-
Mild spasticity	-
Diabetes mellitus	-
Dental crowding/ hypodontia/ small roots/ higharched palate	-
LVH/congenital heart diseases	-
Hepatic fibrosis	-

Recent researchers deduced that renal hypoplasia can be present in these patients without any significant complaint [8]. Our patient was diagnosed with urethral stricture; for which cystostomy was performed. Renal impairment is a leading cause of mortality in these patients and needs to be addressed appropriately [2].

LMBBS patients are generally friendly and happy people. To ensure the quality of life, pediatrician, ophthalmologist, nephrologist, pathologist, cardiologist, and other health care personnel should be kept in the loop with the patient's condition. Frequent BP and ophthalmological assessment and yearly renal function and lipid profile evaluation should be done [9]. Genetic counseling should be offered to the family guiding them about the risk of this condition in future offspring as well as complications that the other siblings carrying even one mutated gene need to be evaluated for like hypertension and renal impairment [10].

This instance is being reported for its rarity; and the presence of abdominal distension with LMBBS has not been reported in the literature as yet, to the best of our comprehension.

## Conclusions

LMBBS is a disorder with an identified pentad of symptoms which are obesity, hypogonadism, intellectual impairment, polydactyly and retinitis pigmentosa. Renal function loss is identified as the most common cause of mortality in these patients. Because of the seemingly unrelated symptoms, the disorder remains largely underdiagnosed. Emphasis should be laid on spreading awareness about this condition as well-rounded care by different specialties and serial laboratory follow up can ensure a good quality of life in these individuals. Furthermore, genetic counseling of the affected families could not only warn them of the possibility of having more children in future with the said condition but also raise awareness about the need to get the other family members assessed for renal and cardiovascular problems.
